# Supplementary material for: Transcriptome-wide association analyses reveal the impact of regulatory variants on rice panicle architecture and causal gene regulatory networks
Source: Nat Commun. 2023 Nov 18;14:7501. doi: 10.1038/s41467-023-43077-6 (PMC10657423; doi:10.1038/s41467-023-43077-6)
Supplement: Supplementary file 1 — Supplementary Information [file 41467_2023_43077_MOESM1_ESM.pdf]

Transcriptome-wide association analyses reveal the impact of regulatory variants on rice panicle architecture and causal gene regulatory networks

Ming *et al.*

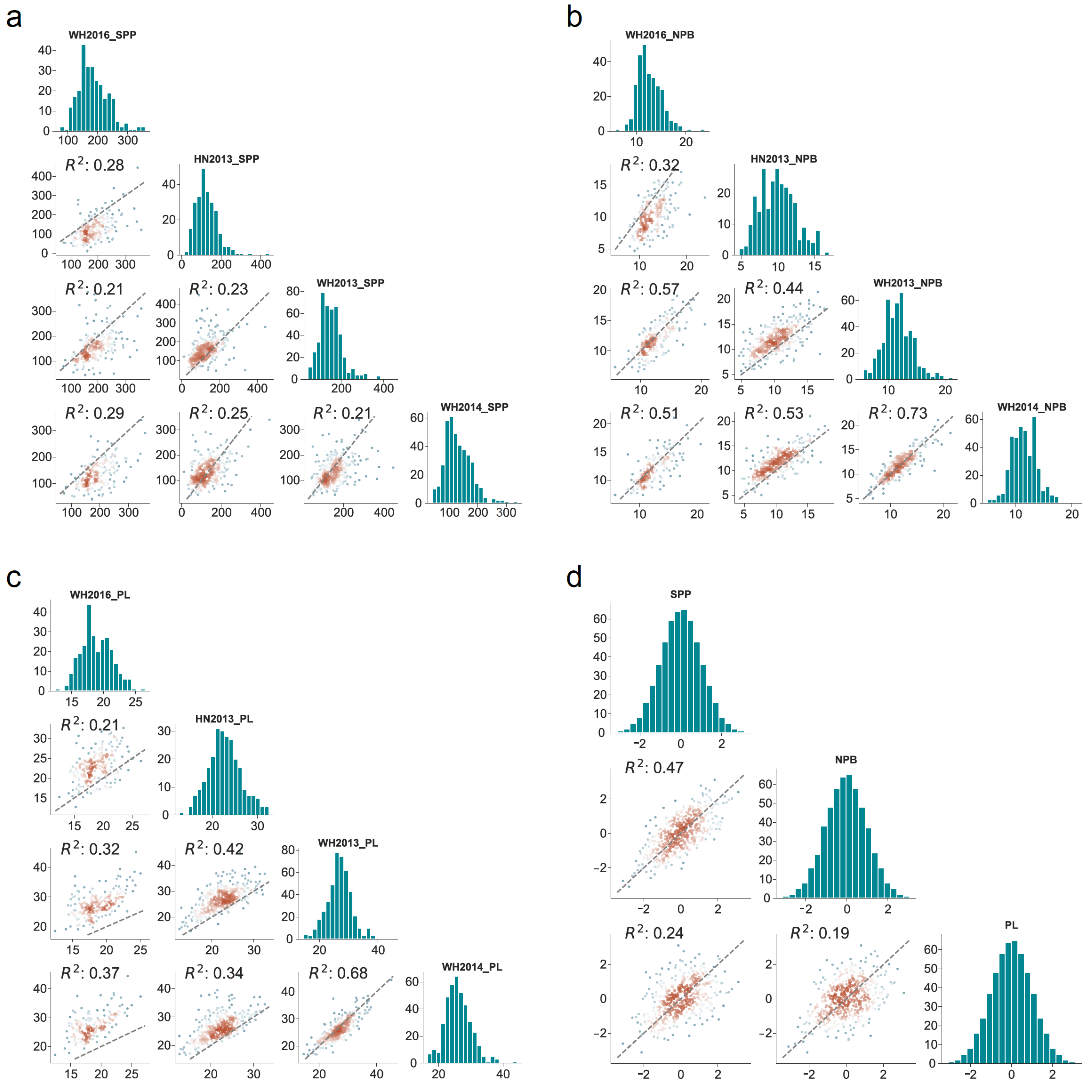

Supplementary Figure 1: Correlation between panicle phenotypes. (a-c) Phenotypic correlations in different years and locations for spikelets per panicle (SPP), number of primary branches (NPB), and panicle length (PL). The R^2 is the square of Pearson's correlation coefficient. The histograms on the diagonal show the distribution of phenotypes. WH2016, HN2013, WH2013, and WH2014 indicate the field trials performed in Wuhan in 2016, Hainan in 2013, Wuhan in 2013, and Wuhan in 2014, respectively. **(d)** Correlations of BLUP value between SPP, NPB, and PL. Source data are provided as a Source Data file.

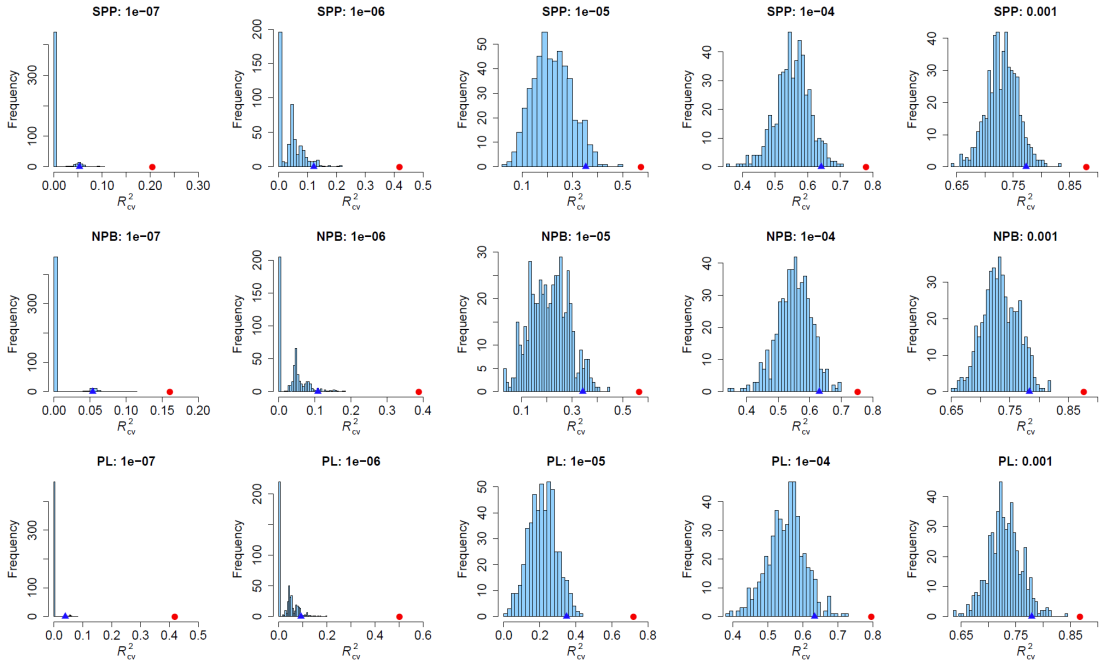

Supplementary Figure 2: Histograms of the variance of observed and permuted panicle phenotypes explained by GWAS lead variants identified at different *P*-value thresholds in 529 varieties. Blue triangles indicate the 0.95 quantiles of explained variance of the 500 permuted panicle traits, and red dots indicate explained variance of the observed panicle traits. The explained phenotypic variances were assessed by tenfold cross-validation (R^2 of predicted values versus observed or permuted phenotypes) using LASSO (see Methods).

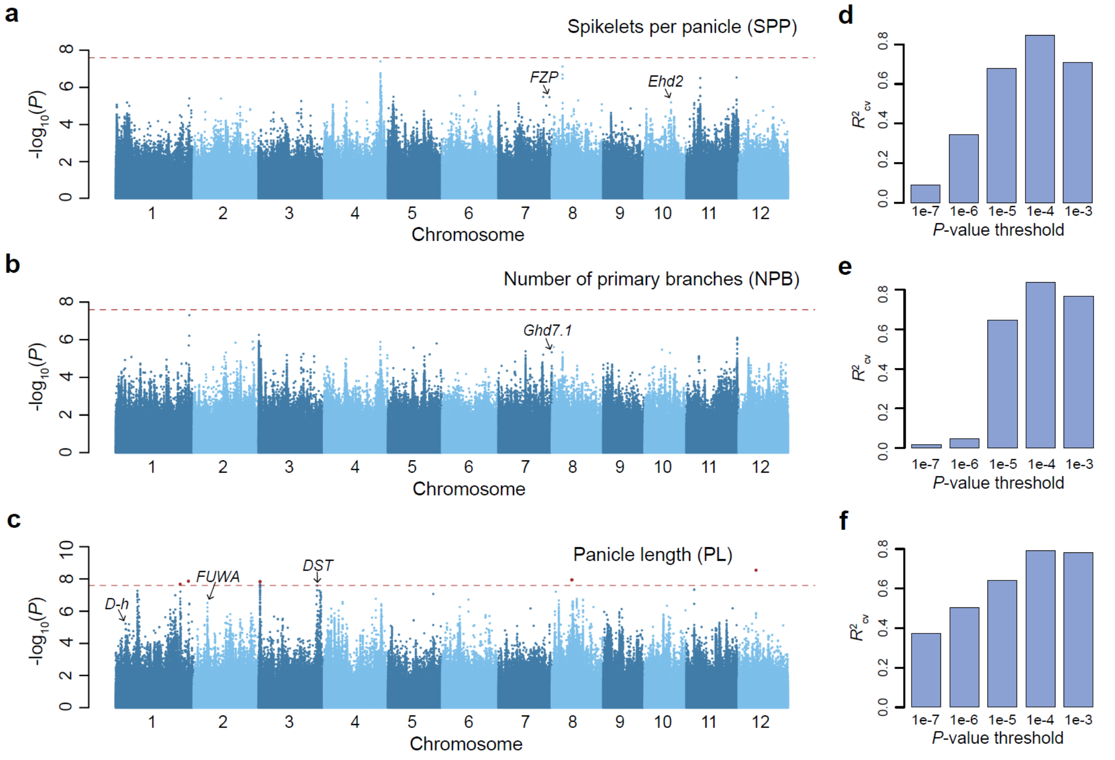

Supplementary Figure 3: GWAS and genetic variance composition of panicle traits in 275 varieties. (a-c) Manhattan plots of GWAS for SPP, NPB, and PL in 275 varieties with transcriptome data. The red dashed lines indicate the genome-wide significance threshold (2.54×10^{-8}), and the red points indicate lead variants of genome-wide significant QTLs. Genes that have been reported to be related to panicle architecture within 100 kb of significant loci at the 1×10^{-5} threshold are marked out. **(d-f)** The phenotypic variance explained by GWAS lead variants identified at different *P*-value thresholds in 275 varieties for SPP, NPB, and PL. The explained phenotypic variances are assessed by tenfold cross-validation using LASSO. R^2: predicted values versus observed phenotypes.

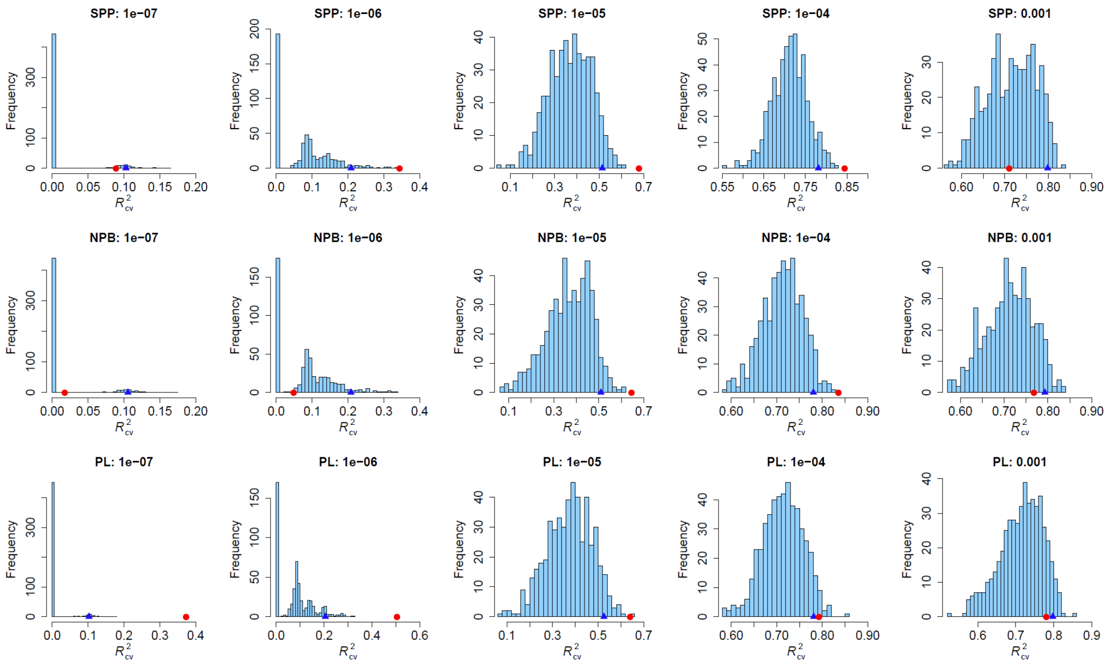

Supplementary Figure 4: Histograms of the variance of observed and permuted panicle phenotypes explained by GWAS lead variants identified at different *P*-value thresholds in 275 varieties. Blue triangles indicate the 0.95 quantiles of explained variance of the 500 permuted panicle traits, and red dots indicate explained variance of the observed panicle traits. The explained phenotypic variances were assessed by tenfold cross-validation (R^2 of predicted values versus observed or permuted phenotypes) using LASSO.

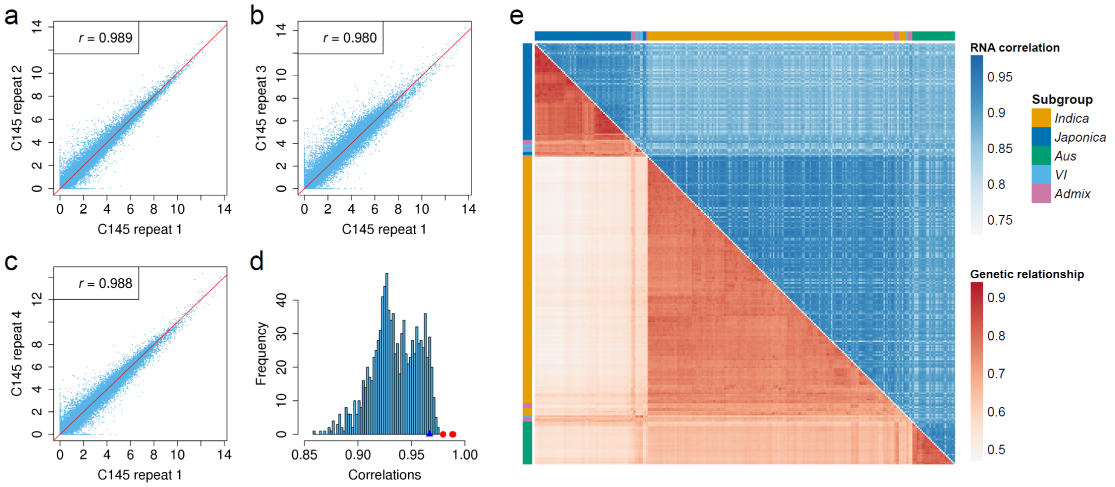

Supplementary Figure 5: RNA level consistency among biological replicates and diversity among varieties. (a-c) RNA level correlations between biological replicates of Zhenshan 97 (C145). The values are TPMs plus 1 and then log2 transformed, and the r is Pearson's correlation coefficient between two replicates. **(d)** Histogram of RNA level correlations of 1000 randomly selected pairs of varieties. The blue triangle indicates the 0.95 quantiles of these 1000 correlation coefficients, and the red dots indicate correlation coefficients between biological replicates of Zhenshan 97 (C145). **(e)** Genetic relationships (lower triangle) and RNA-level correlations (upper triangle) among 275 varieties.

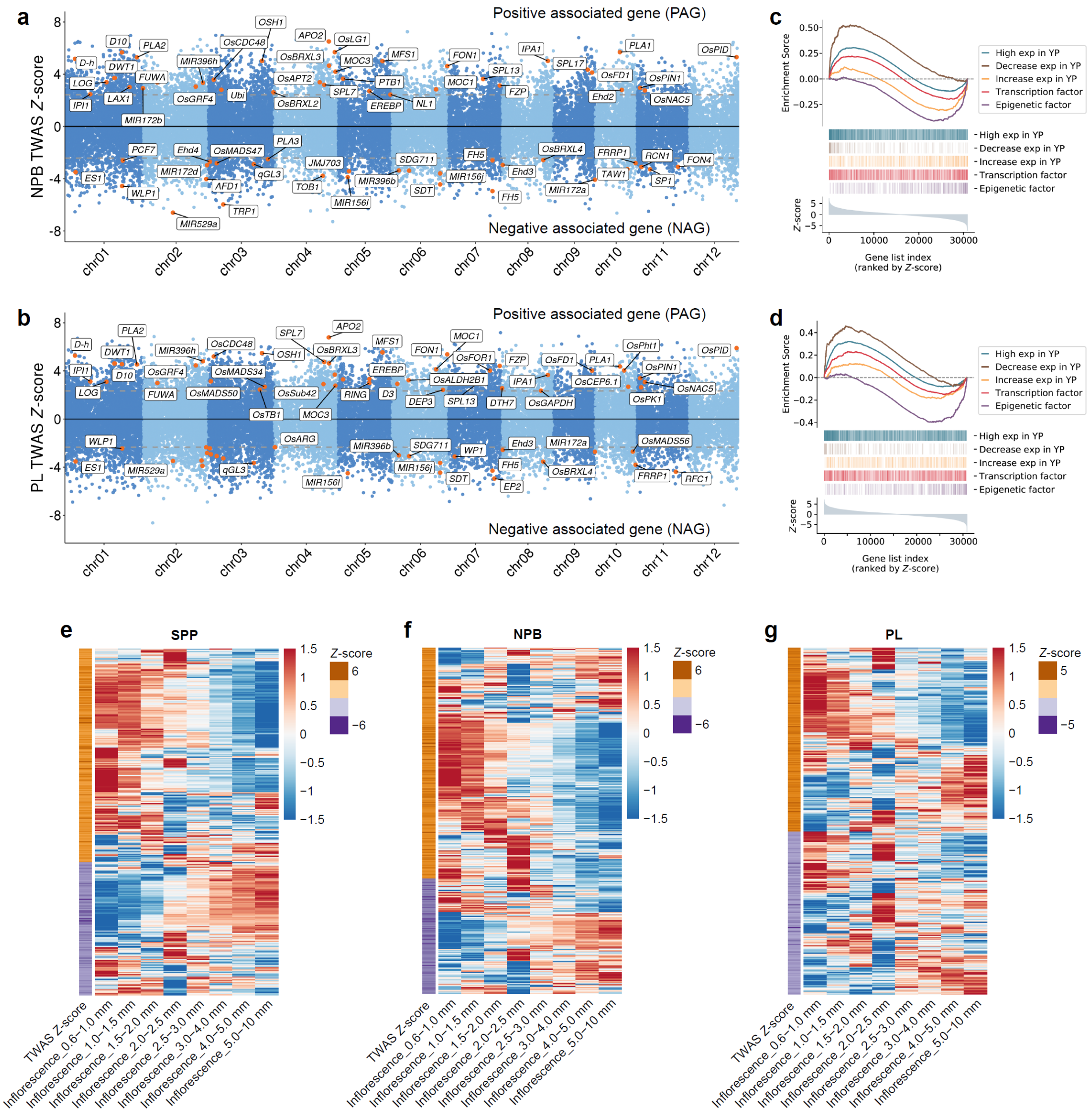

Supplementary Figure 6: TWAS and the characteristics of TWAS significant genes, related to Figure 3. (a and b) Manhattan plots of TWAS for NPB and PL, as in Figure 3a. **(c and d)** GSEA for TWAS significant genes of NPB and PL, as in Figure 3b. **(e-g)** Expression profiles in young panicles for TWAS significant genes (top 500) of SPP, NPB, and PL, respectively. Each row is a gene. The expression levels of each gene were normalized. The expression data were obtained from RiceXPro.

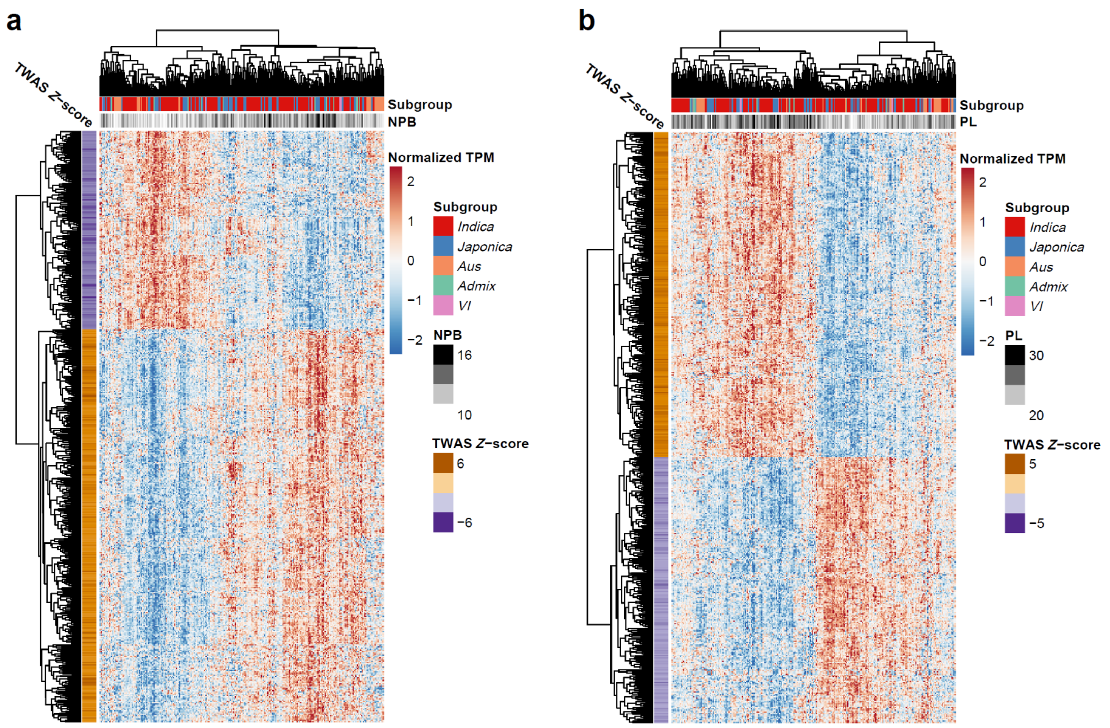

Supplementary Figure 7: Expression patterns of NPB (a) and PL (b) top 500 TWAS significant genes in the 275 varieties. Each column is a variety, and each row is a gene**.**

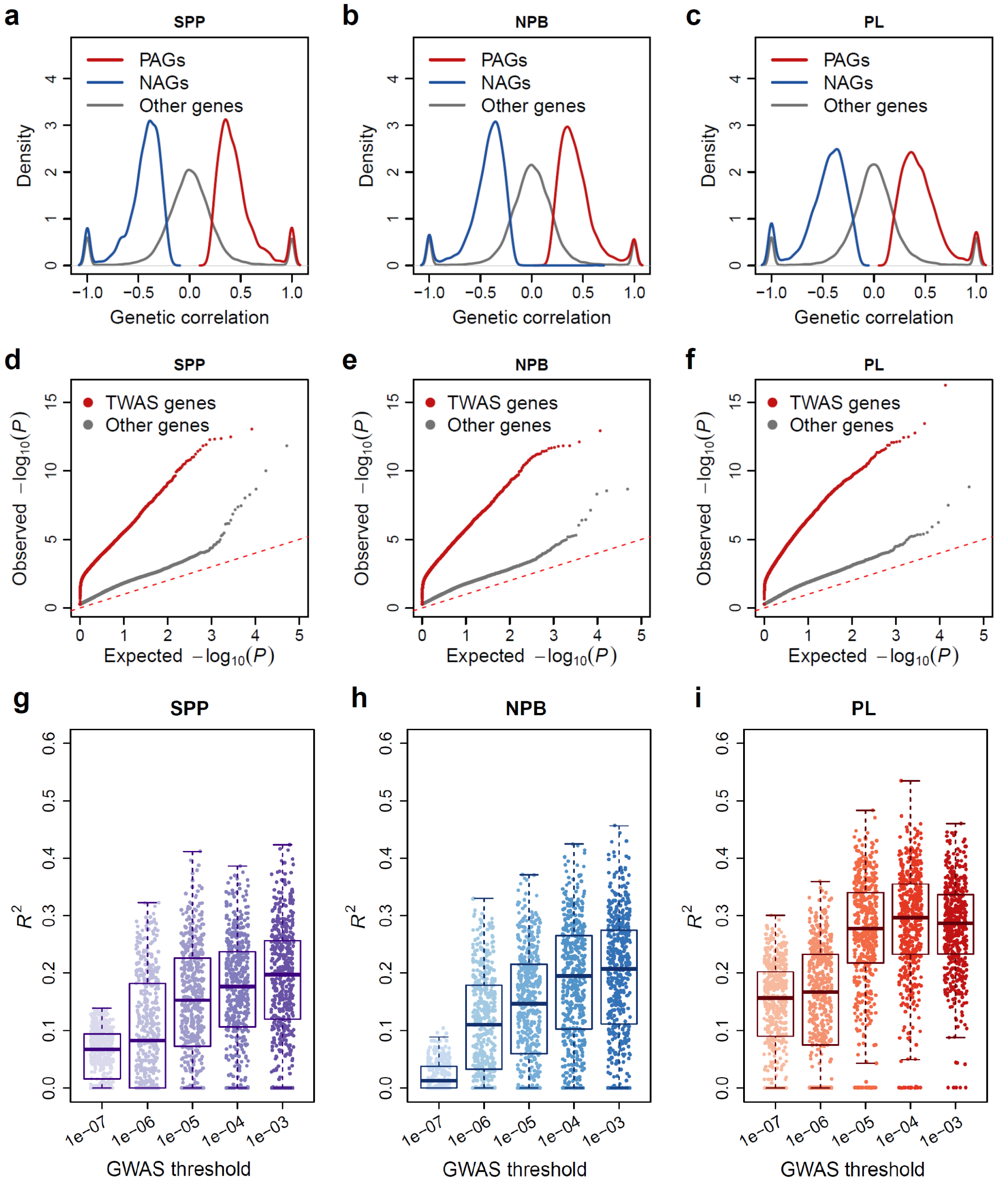

Supplementary Figure 8: Genetic correlations between panicle traits and TWAS significant genes. (a-c) The distribution of genetic correlations between phenotypes and the expression of TWAS significant genes for SPP (a), NPB (b), and PL (c), respectively. Genetic correlations were estimated using GCTA (see Methods). **(d-f)** Q-Q plots showing the *P*-values of the genetic correlations between phenotypes and TWAS significant genes for SPP (d), NPB (e), and PL (f), respectively. **(g-i)** R^2: the square of the Pearson correlation coefficient between the panicle trait and the predicted expression values of the top 500 TWAS significant genes of SPP (g), NPB (h), and PL (i), respectively. Each point represents a gene. The predicted expression values are predicted using the LASSO model based on lead variants of GWAS QTLs identified at different thresholds in the 529 varieties' panel. The definitions of the box plots are the same as in Figures 4b-d. Source data are provided as a Source Data file.

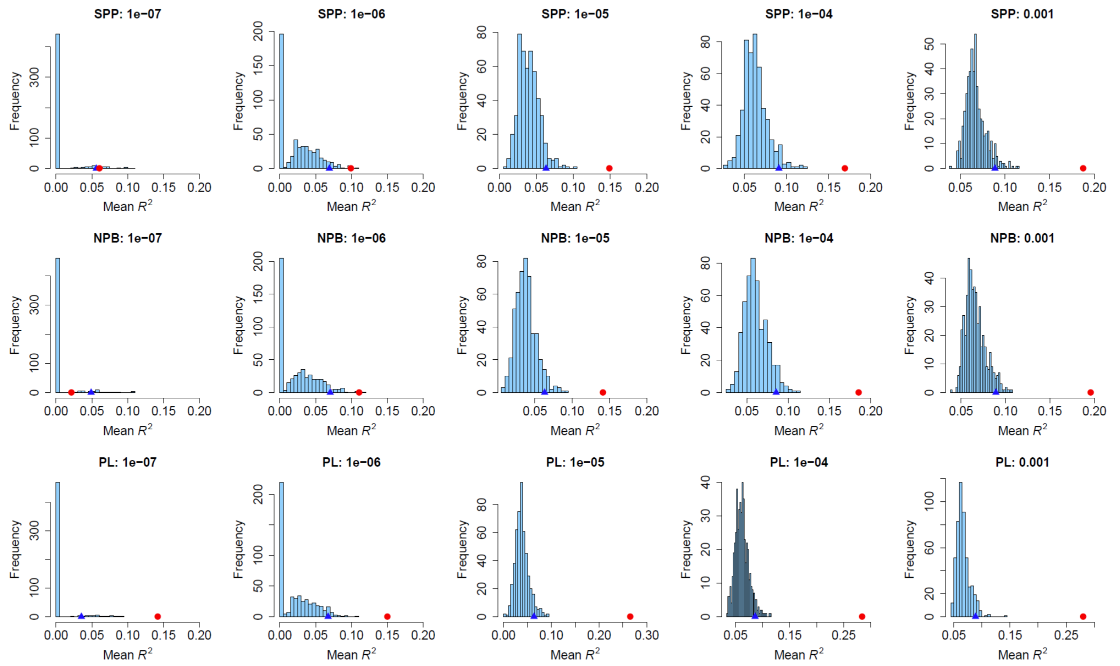

Supplementary Figure 9: Histograms of the mean of squared Pearson correlation coefficient between predicted expression of TWAS significant genes (top 500) and phenotypes for panicle traits and their 500 permuted traits. Blue triangles indicate the 0.95 quantiles of the mean of squared Pearson correlation coefficient for the 500 permuted traits, and red dots indicate the mean of squared Pearson correlation coefficient for panicle trait. Gene expressions were predicted using GWAS lead variants identified at different thresholds in the 529 varieties' panels, with the LASSO model (see Methods).

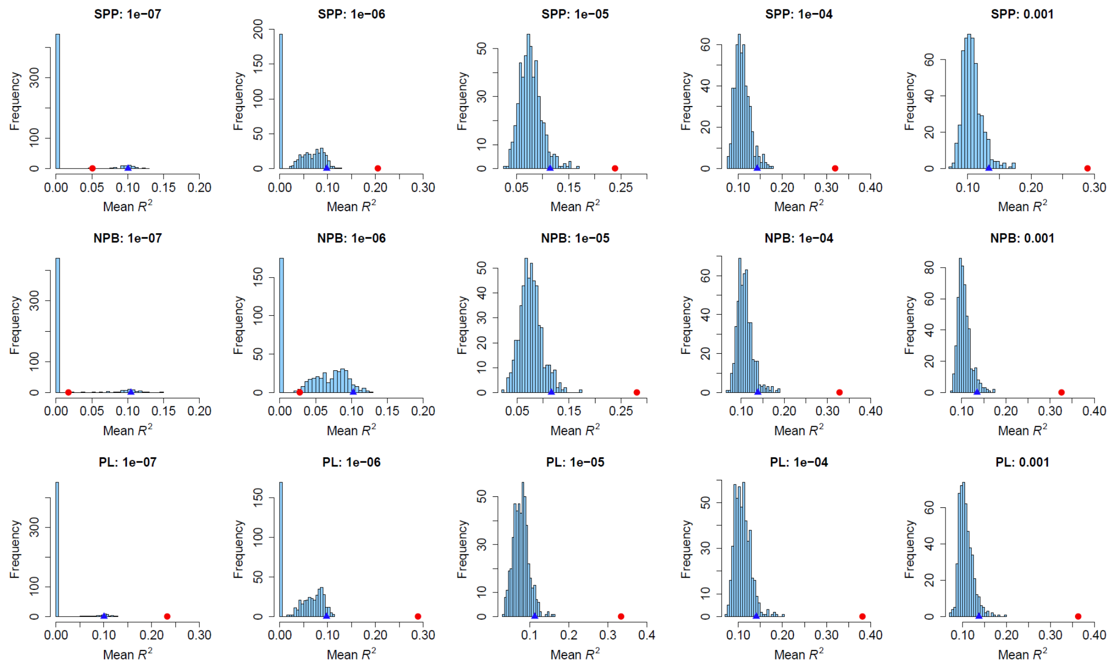

Supplementary Figure 10: Histograms of the mean of squared Pearson correlation coefficient between predicted expression of TWAS significant genes (top 500) and phenotypes for panicle traits and their 500 permuted traits. Blue triangles indicate the 0.95 quantiles of the mean of squared Pearson correlation coefficient for the 500 permuted traits, and red dots indicate the mean of squared Pearson correlation coefficient for panicle trait. Gene expressions were predicted using GWAS lead variants identified at different thresholds in the 275 varieties' panels, with the LASSO model.

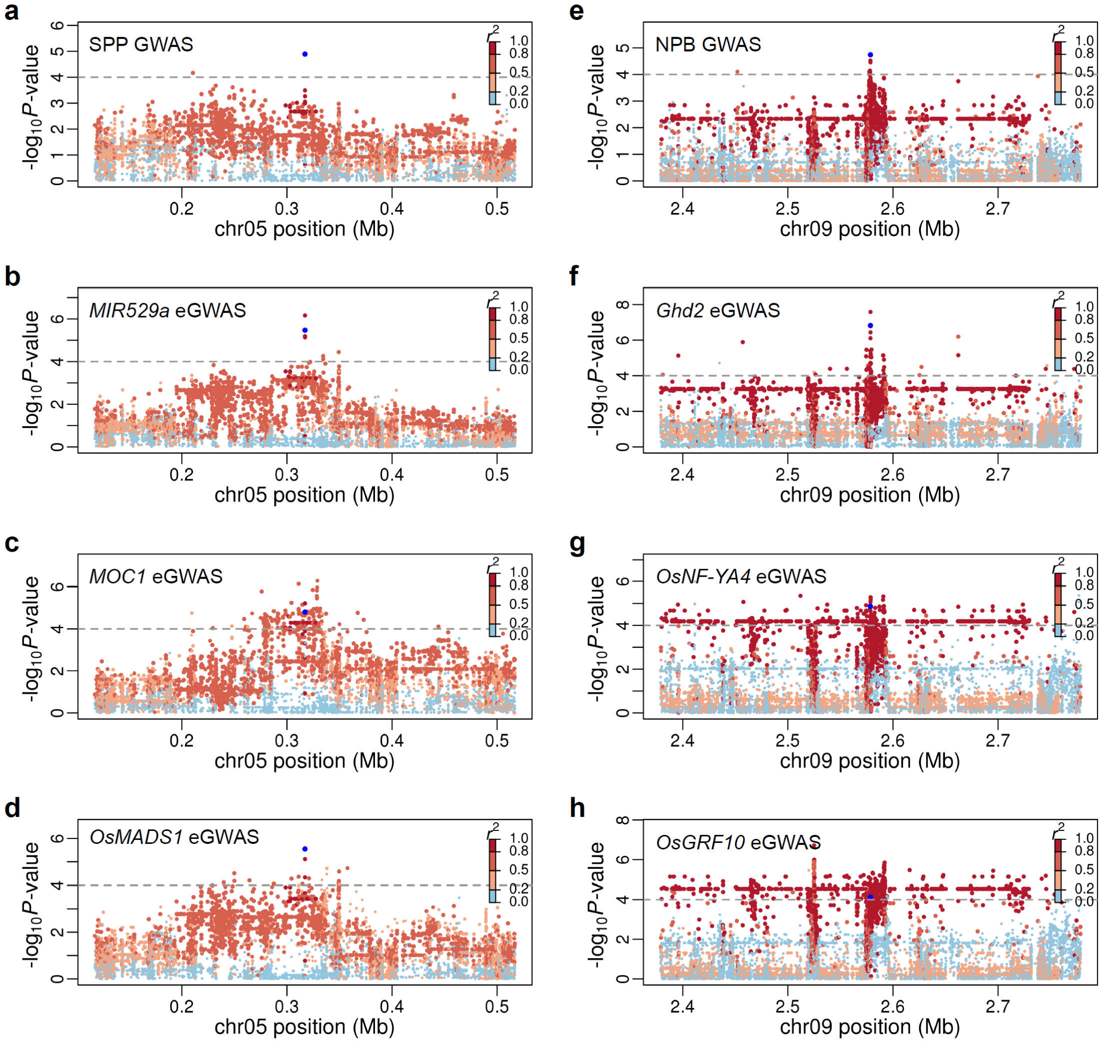

Supplementary Figure 11: Examples of pQTL-eQTL hotspots and their target genes. (a-d) Regional association plots of GWAS of SPP and eGWAS of several target genes, 400 kb window centered on the hSPP.05.1. The association -log10 *P*-values (y-axis) are plotted against variant positions (x-axis). The colors of the dots represent the LD r^2 of each variant with the GWAS lead variant (dark blue). **(e-h)** Regional association plots of GWAS of NPB and eGWAS of several target genes, 400 kb window centered on the hNPB.09.1.

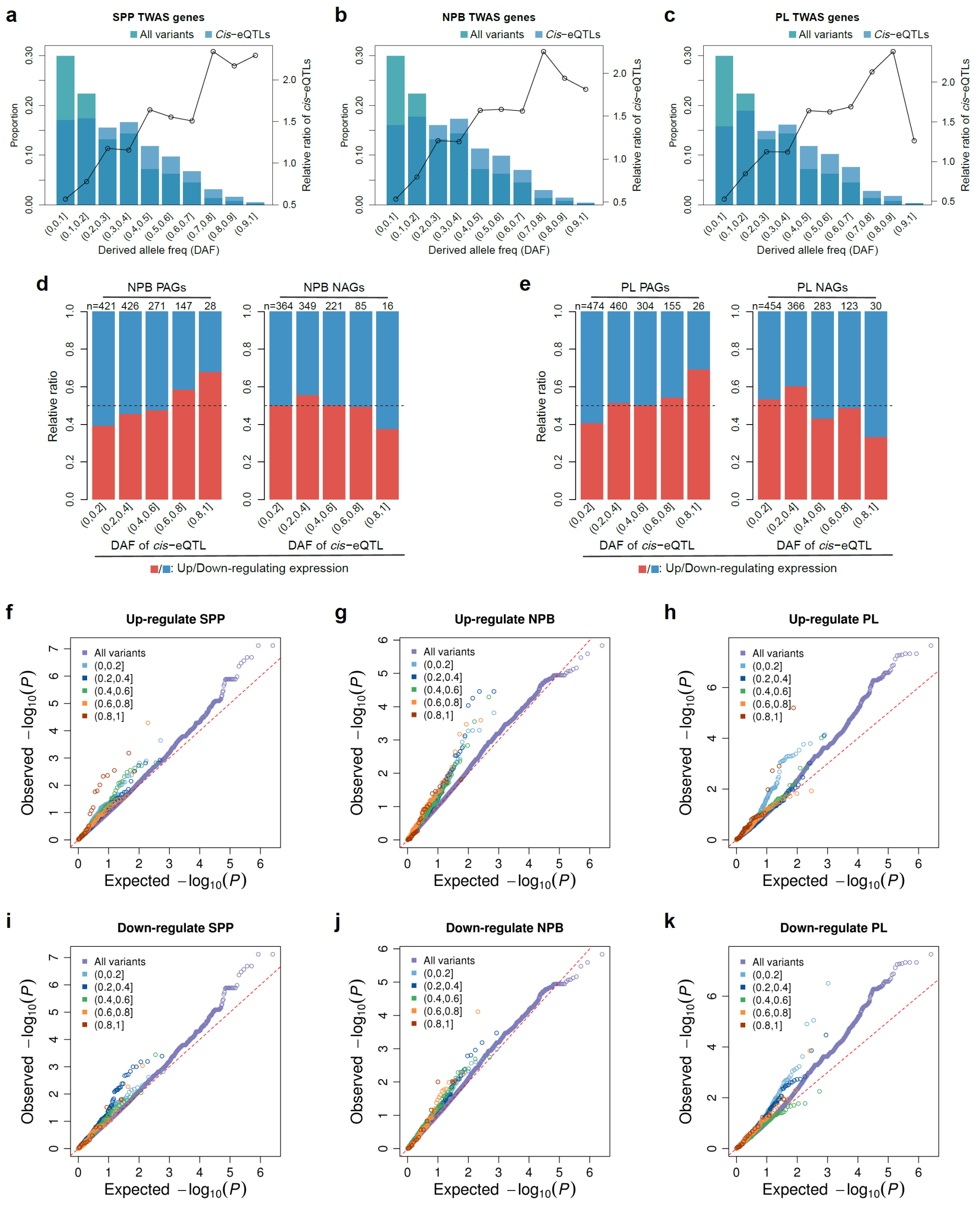

Supplementary Figure 12: Impact of derived alleles on the expression of TWAS significant gene and phenotype of panicle traits, related to Figure 5. (a-c) The relative ratio of the proportion of *cis*-eQTLs to the proportion of all variants in each DAF interval. The cis-eQTLs were from the TWAS significant genes of SPP (a), NPB (b), and PL (c), respectively. As in Figure 5a. **(d and e)** The impact of derived alleles on the expression of TWAS-significant genes of NPB (d) and PL (e), as in Figure 5b. **(f-h)** GWAS signal Q-Q plot of *cis*-eQTLs for TWAS significant genes whose derived allele of *cis*-eQTL may have a positive effect on phenotype (up-regulate PAGs or down-regulate NAGs). TWAS significant genes were divided into different groups according to the DAF of *cis*-eQTL and indicated by different colors. **(i-k)** GWAS signal Q-Q plot of *cis*-eQTLs for TWAS significant genes whose derived allele of *cis*-eQTL may have a negative effect on phenotype (up-regulate NAGs or down-regulate PAGs). TWAS significant genes were divided into different groups according to the DAF of *cis*-eQTL and indicated by different colors. Source data underlying Supplementary Fig. 12a-e are provided as a Source Data file.

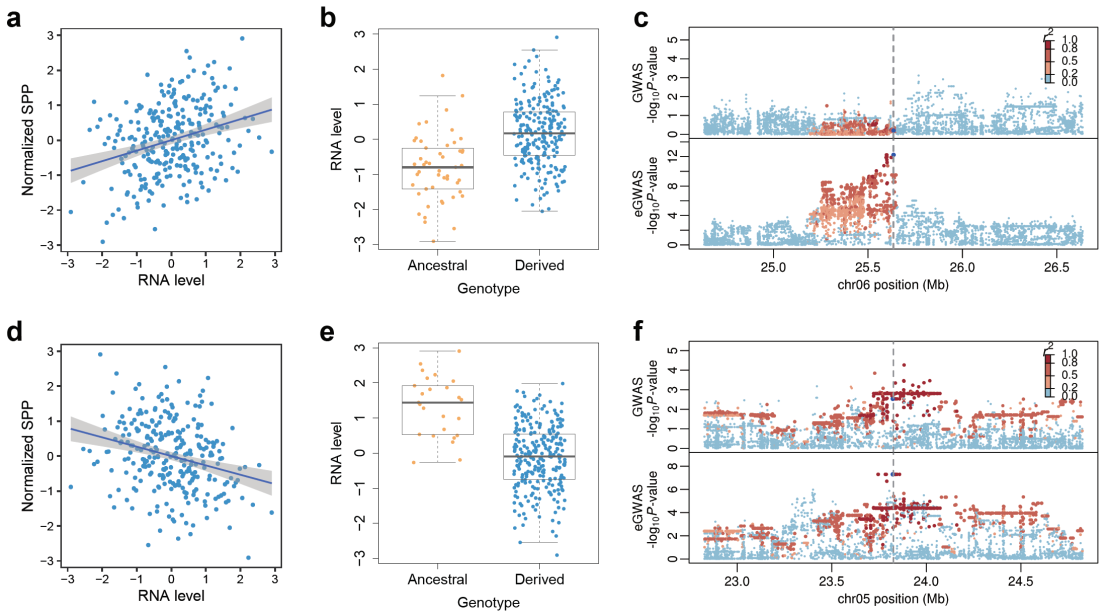

Supplementary Figure 13: Examples of TWAS significant genes whose cis-regulatory variants may be subject to selection. (a) Correlation between RNA levels of LOC_Os06g42630 and SPP. **(b)** RNA levels of ancestral and derived alleles for the *cis*-eQTL (vg0625635377) of LOC_Os06g42630. The center line of the box plot indicates the median. **(c)** Regional association plot of SPP GWAS (top) and LOC_Os06g42630 eGWAS (bottom), 2 Mb window centered on LOC_Os06g42630. The association -log10 *P*-values (y-axis) are plotted against variant positions (x-axis). The colors of the dots represent the LD r^2 of each variant with the lead variant of *cis*-eQTL (dark blue). **(d)** Correlation between RNA levels of LOC_Os05g40630 and SPP. **(e)** RNA levels of ancestral and derived alleles for the *cis*-eQTL (vg0523822557) of LOC_Os05g40630. The center line of the box plot indicates the median. **(f)** Regional association plot of SPP GWAS (top) and LOC_Os05g40630 eGWAS (bottom), 2 Mb window centered on LOC_Os05g40630. The association -log10 *P*-values (y-axis) are plotted against variant positions (x-axis). The colors of the dots represent the LD r^2 of each variant with the lead variant of *cis*-eQTL (dark blue). Source data underlying Supplementary Fig. 13a, b, d, e are provided as a Source Data file.

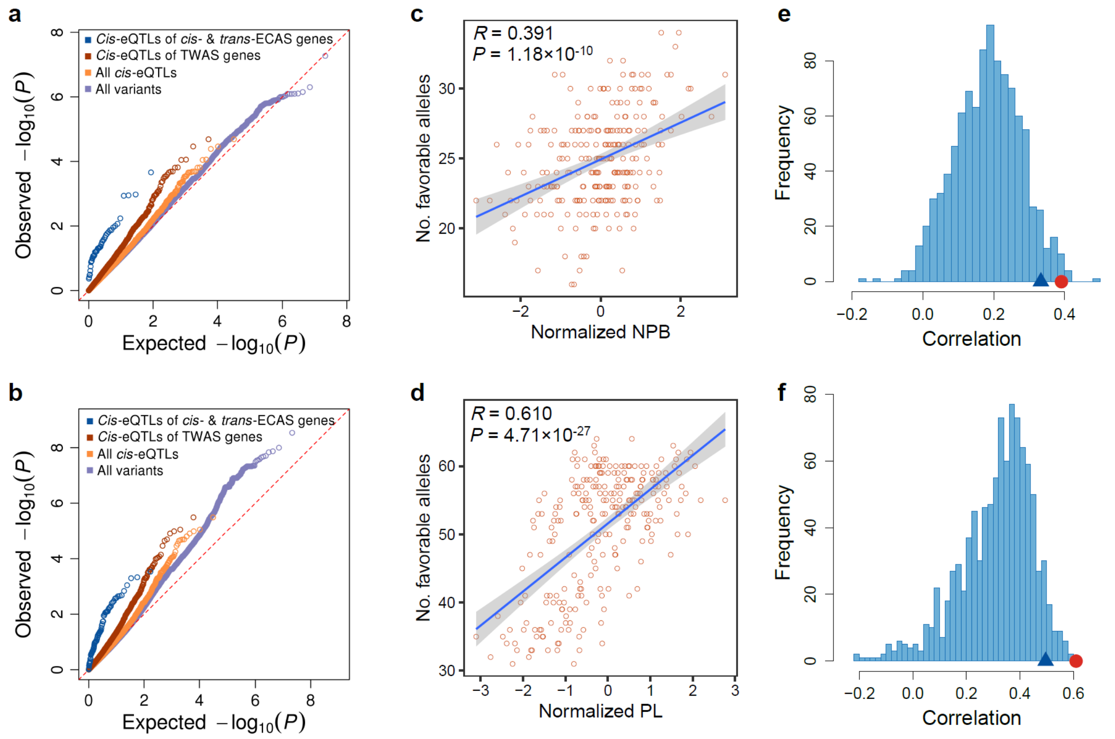

Supplementary Figure 14: GWAS signal distribution and predictive power of *cis*-eQTLs of *cis*- & *trans*-ECAS genes. (a and b) GWAS signal Q-Q plot of *cis*-eQTLs for *cis*- & *trans*-ECAS genes of NPB (a) and PL (b), as in Figure 6g. **(c and d)** The correlations between phenotypes and the number of favorable alleles of the *cis*-eQTLs for the *cis*- & *trans*-ECAS genes of NPB (c) and PL (d), as in Figure 6h. **(e and f)** Histogram of the correlations between phenotype and the number of favorable alleles at *cis*-eQTLs of randomly selected TWAS significant genes, for NPB (e) and PL (f), as in Figure 6i. Source data underlying Supplementary Fig. 14c, d are provided as a Source Data file.

a

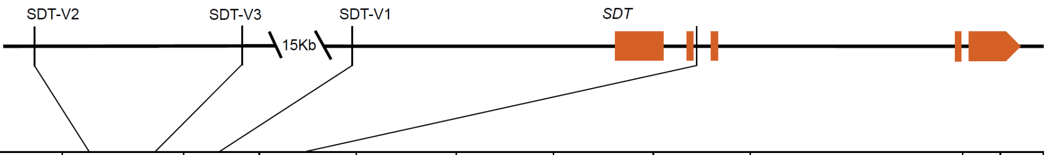

b

Haplotype	Upstream		Promoter	Intron	SDT expression log2(TPM+1)	P-value of expression difference (Compared to Type3)	SPP	P-value of SPP difference (Compared to Type3)	Subpopulation					All	Wild rice
	-23786	-20371	-4339	(1335, 1399)					IND	TEJ	TRJ	Aus	Others		
Type1	A	A	A	Ref	3.71±0.80	2.05×10^{-12}	136.98±33.06	5.83×10^{-4}	0	10	3	0	3	16	-
Type2	G	G	A	Ref	1.91±0.77	2.75×10^{-11}	146.18±28.97	2.72×10^{-4}	163	11	3	27	21	225	126
Type3	G	G	G	~2.7 Kb CTAT repeat Insertion	0.73±0.54	–	179.82±38.00	–	6	1	15	0	2	24	1

c

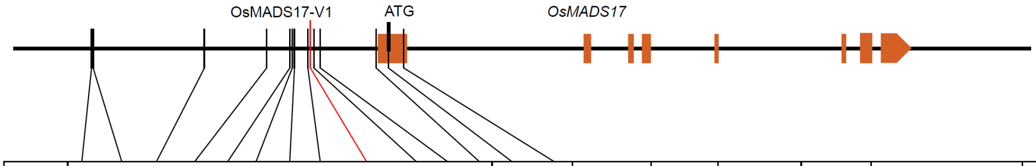

d

Haplotype	Promoter												CDS		OsMADS17 expression log2(TPM+1)	P-value of expression difference (Compared to Type1)	SPP	P-value of SPP difference (Compared to Type1)	Subpopulation					All	Wild rice
	-3154	-3132	-1916	-1226	-975	-938	-920	-761	(-759, -721)	-710	-636	-14	122	289					IND	TEJ	TRJ	Aus	Others		
Type1	A	C	C	C	C	A	T	C	Ref	G	T	A	A	T	4.98±0.63	–	137.60±24.98	–	8	17	2	8	12	47	-
Type2	G	C	G	C	C	A	T	T	11bp DEL	G	C	A	65bp Insertion	C	4.34±0.67	7.94×10^{-8}	147.59±32.10	0.030	130	0	0	7	3	140	-
Type3	G	T	G	T	T	G	TAA AA	N	39bp DEL	A	C	C	65bp Insertion	C	3.95±0.5	4.45×10^{-12}	159.12±32.14	1.86×10^{-3}	2	4	12	6	10	34	-

Supplementary Figure 15: Haplotype analysis of *SDT and OsMADS17*. (a) Schematic of the gene structure of *SDT*. Vertical lines indicate the variants significantly associated (*P*-value < 10^{-5}) with *SDT* expression. **(b)** Haplotype analysis of the *SDT* gene region in 275 cultivated rice with young panicle transcriptome data and in 135 wild rice. The data of *SDT* expression and spikelet per panicle are means ± SD. SDT-V1, SDT-V2, and SDT-V3 were used to count the frequency of the three types in wild rice. Comparisons are made by two-tailed Student's *t*-test. IND *indica* population, TEJ temperate *japonica* population, TRJ tropical *japonica* population. **(c)** Schematic of the gene structure of *OsMADS17*. Vertical lines indicate variants significantly associated (*P*-value < 10^{-5}) with *OsMADS17* expression (5 kb upstream of TSS to TSS) or variants located in the *OsMADS17* coding region. **(d)** Haplotype analysis of the *OsMADS17* gene region in 275 cultivated rice with young panicle transcriptome data and in 135 wild rice. The data of *OsMADS17* expression and spikelet per panicle are means ± SD. Comparisons are made by two-tailed Student's *t*-test. IND *indica* population, TEJ temperate *japonica* population, TRJ tropical *japonica* population. Source data underlying Supplementary Fig. 15b, d are provided as a Source Data file.

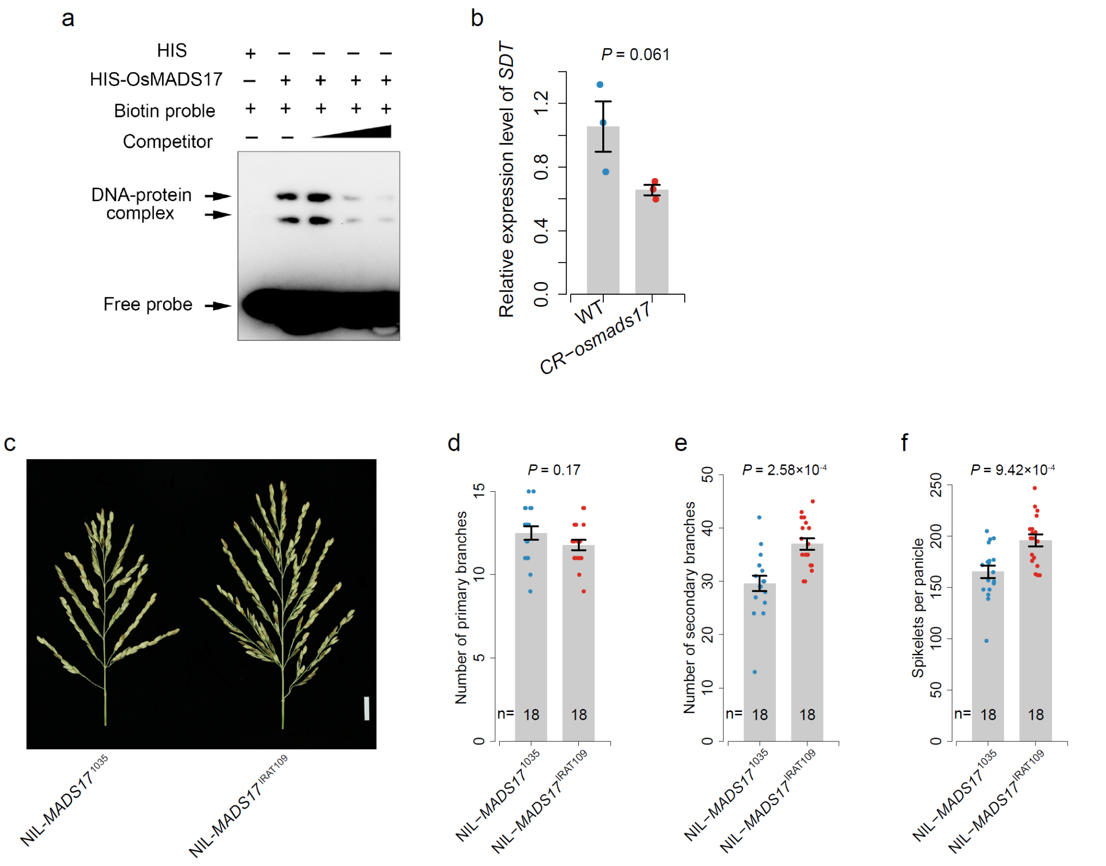

Supplementary Figure 16: Experimental validation of *OsMADS17* regulating *SDT* transcription and breeding application of *OsMADS17*. (a) EMSA of *OsMADS17* directly binding to the dOCR upstream of *SDT*. **(b)** Relative expression levels of *SDT* in young panicles (3 mm) of *CR-osmads17* mutant and WT (n = 3 replicates). Data are means ± SEM. Comparisons are made by one-tailed Student's *t*-test. **(c)** Panicle morphologies of NIL-*MADS17*^{1035} and NIL-*MADS17*$^{\mathrm{IRAT109}}$. NIL-*MADS17*^{1035} has the reference genotype on OsMADS17-V1, while NIL-*MADS17*$^{\mathrm{IRAT109}}$ has the 39-bp deletion on OsMADS17-V1. Scale bar, 2 cm. **(d-f)** Quantification of the number of primary branches (d), number of secondary branches (e), and spikelets per panicle (f) in the main panicle of NIL-*MADS17*^{1035} and NIL-*MADS17*$^{\mathrm{IRAT109}}$. Data are means ± SEM. Comparisons are made by two-tailed Student's *t*-test. Source data underlying Supplementary Fig. 16a, b, d-f are provided as a Source Data file.
