# Supplementary material for: Transcriptome-wide association analyses reveal the impact of regulatory variants on rice panicle architecture and causal gene regulatory networks
Source: Nat Commun. 2023 Nov 18;14:7501. doi: 10.1038/s41467-023-43077-6 (PMC10657423; doi:10.1038/s41467-023-43077-6)
Supplement: Supplementary file 3 — Description of Additional Supplementary Files [file 41467_2023_43077_MOESM3_ESM.pdf]

Description of Additional Supplementary Files

Supplementary Data 1: Panicle phenotype in Wuhan 2016.

Supplementary Data 2: Known panicle development-related genes located in the vicinity of the QTL identified at the threshold of 1e-5.

Supplementary Data 3: Summary of accessions for RNA sequencing.

Supplementary Data 4: Summary of SPP TWAS significant genes.

Supplementary Data 5: Summary of NPB TWAS significant genes.

Supplementary Data 6: Summary of PL TWAS significant genes.

Supplementary Data 7: Summary of GSEA results of TWAS significant genes.

Supplementary Data 8: Summary of GO enrichment results of TWAS significant genes.

Supplementary Data 9: Summary of transcription factor families enriched in TWAS significant genes.

Supplementary Data 10: Summary of enriched TF binding motifs of TWAS significant genes.

Supplementary Data 11: Summary of pQTL-eQTL hotspots of TWAS significant genes.

Supplementary Data 12: Summary of *cis*- & *trans*-ECAS significant genes.

Supplementary Data 13: Summary of significant associations between SPP TWAS-significant genes (FDR < 0.01) and the *cis*- and *trans*-EC of *OsMADS17* and *SDT*.

Supplementary Data 14: List of primers used in experiments.
