# Supplementary material for: Transcriptome-wide association analyses reveal the impact of regulatory variants on rice panicle architecture and causal gene regulatory networks
Source: Nat Commun. 2023 Nov 18;14:7501. doi: 10.1038/s41467-023-43077-6 (PMC10657423; doi:10.1038/s41467-023-43077-6)
Supplement: Supplementary file 5 — Reporting Summary [file 41467_2023_43077_MOESM5_ESM.pdf]

nature portfolio

Corresponding author(s): Weibo Xie, Changyin Wu

Last updated by author(s): Oct 16, 2023

Reporting Summary

Nature Portfolio wishes to improve the reproducibility of the work that we publish. This form provides structure for consistency and transparency in reporting. For further information on Nature Portfolio policies, see our Editorial Policies and the Editorial Policy Checklist.

Statistics

For all statistical analyses, confirm that the following items are present in the figure legend, table legend, main text, or Methods section.

n/a	Confirmed	
☐	☒	The exact sample size (*n*) for each experimental group/condition, given as a discrete number and unit of measurement
☐	☒	A statement on whether measurements were taken from distinct samples or whether the same sample was measured repeatedly
☐	☒	The statistical test(s) used AND whether they are one- or two-sided<br>*Only common tests should be described solely by name; describe more complex techniques in the Methods section.*
☐	☒	A description of all covariates tested
☐	☒	A description of any assumptions or corrections, such as tests of normality and adjustment for multiple comparisons
☐	☒	A full description of the statistical parameters including central tendency (e.g. means) or other basic estimates (e.g. regression coefficient) AND variation (e.g. standard deviation) or associated estimates of uncertainty (e.g. confidence intervals)
☐	☒	For null hypothesis testing, the test statistic (e.g. *F*, *t*, *r*) with confidence intervals, effect sizes, degrees of freedom and *P* value noted<br>*Give P values as exact values whenever suitable.*
☒	☐	For Bayesian analysis, information on the choice of priors and Markov chain Monte Carlo settings
☒	☐	For hierarchical and complex designs, identification of the appropriate level for tests and full reporting of outcomes
☐	☒	Estimates of effect sizes (e.g. Cohen's *d*, Pearson's *r*), indicating how they were calculated

Our web collection on statistics for biologists contains articles on many of the points above.

Software and code

Policy information about availability of computer code

Data collection	RNA-seq libraries were sequenced using the Illumina HiSeqX platform; The relative luciferase activity was measured using the DLR assay system (Promega) and the TECAN Infinite M200 microplate reader; qRT-PCR was performed with the Applied Biosystems 7500 real-time PCR detection system using SYBR Green MasterMix (Applied Biosystems).
Data analysis	Salmon (version 0.8.0); PEER (version 1.0); lme4 (version 1.1.27.1); PLINK (version 1.90b4); EMMAX (cpgen v0.1, https://rdrr.io/cran/cpgen/); GEC (version 0.2); glmnet (version 4.1.2); GCTA (version 1.93.2beta); GSEApy (version 0.9.16). The usage and parameters of the software are described in the manuscript. The codes for identifying causal genes affecting phenotypes and for constructing regulatory networks of functional genes have been deposited in Zenodo (https://doi.org/10.5281/zenodo.10004834) and in Github (https://github.com/Minglc/CisTrans-ECAS)

For manuscripts utilizing custom algorithms or software that are central to the research but not yet described in published literature, software must be made available to editors and reviewers. We strongly encourage code deposition in a community repository (e.g. GitHub). See the Nature Portfolio guidelines for submitting code & software for further information.

Data

Policy information about availability of data

All manuscripts must include a data availability statement. This statement should provide the following information, where applicable:

- Accession codes, unique identifiers, or web links for publicly available datasets
- A description of any restrictions on data availability
- For clinical datasets or third party data, please ensure that the statement adheres to our policy

RNA-seq data generated in this study have been deposited in the Genome Sequence Archive under the BioProject accession number PRJCA012684 (https://ngdc.cncb.ac.cn/bioproject/browse/PRJCA012684). Genotype data, variant impact scores, and subgroup classification of the varieties are available in RiceVarMap V2 (https://ricevarmap.ncpgr.cn/). Rice genome sequence and gene annotation information were obtained from RGAP (http://rice.uga.edu/). Annotation information for microRNAs was obtained from miRbase (https://mirbase.org/). The expression profiles of the entire life cycle are available in CREP (http://crep.ncpgr.cn/) for ZS97 and MH63, and in RiceXPro (https://ricexpro.dna.affrc.go.jp/) or Gene Expression Omnibus (GSE21494, GSE39426, GSE39427, GSE39432) for Nipponbare. The list of transcription factors is available in PlantTFDB (http://planttfdb.gao-lab.org/). The list of chromatin modification-related genes is available in Ensembl BioMarts (http://plants.ensembl.org/biomart/martview/). The genotype data of wild rice germplasm used to identify derived alleles are available at RiceHap3 (http://server.ncgr.ac.cn/RiceHap3/), and the sequencing data are available at European Nucleotide Archive under accession number ERP001143 (https://www.ebi.ac.uk/ena/browser/view/PRJEB2829). Data supporting the findings of this work are available within the paper and its Supplementary Information files. Source data are provided with this paper.

Research involving human participants, their data, or biological material

Policy information about studies with human participants or human data. See also policy information about sex, gender (identity/presentation), and sexual orientation and race, ethnicity and racism.

Reporting on sex and gender	N/A
Reporting on race, ethnicity, or other socially relevant groupings	N/A
Population characteristics	N/A
Recruitment	N/A
Ethics oversight	N/A

Note that full information on the approval of the study protocol must also be provided in the manuscript.

Field-specific reporting

Please select the one below that is the best fit for your research. If you are not sure, read the appropriate sections before making your selection.

☒ Life sciences ☐ Behavioural & social sciences ☐ Ecological, evolutionary & environmental sciences

For a reference copy of the document with all sections, see nature.com/documents/nr-reporting-summary-flat.pdf

Life sciences study design

All studies must disclose on these points even when the disclosure is negative.

Sample size	No statistical methods were used to predetermine the sample size. The sample size was determined based on similar articles previously published by us and other researchers [Tang et al., Mol Plant. 2021, 14: 470-487; Liu et al., Genome Biol. 2020, 21:163].
Data exclusions	No data was excluded from the analysis.
Replication	For population-level RNA-seq, samples from one variety were collected and sequenced four times, yielding highly consistent results (Supplementary Fig. 5a-d). For panicle traits in the population, the main panicles of 5 plants were measured for each variety, exhibiting high correlations with phenotype data from an additional three different years or locations (Supplementary Fig. 1a-c). Transient expression assays and qRT-PCR analyses were successfully replicated three times each.
Randomization	All samples were randomly allocated to experimental groups, and potential batch effects in RNA-seq were corrected for using PEER.
Blinding	The phenotypes of CR-osmads17 mutants, wild-type, and near-isogenic lines were measured by individuals blinded to the genotype information. Blinding was not employed in the molecular experiments due to the inapplicability of the experimental procedures, and no bias introduction is expected.

Reporting for specific materials, systems and methods

We require information from authors about some types of materials, experimental systems and methods used in many studies. Here, indicate whether each material, system or method listed is relevant to your study. If you are not sure if a list item applies to your research, read the appropriate section before selecting a response.

Materials & experimental systems

n/a	Involved in the study
☒	☐ Antibodies
☒	☐ Eukaryotic cell lines
☒	☐ Palaeontology and archaeology
☒	☐ Animals and other organisms
☒	☐ Clinical data
☒	☐ Dual use research of concern
☐	☒ Plants

Methods

n/a	Involved in the study
☒	☐ ChIP-seq
☒	☐ Flow cytometry
☒	☐ MRI-based neuroimaging
